# Citrinin in Foods and Supplements: A Review of Occurrence and Analytical Methodologies

**DOI:** 10.3390/foods10010014

**Published:** 2020-12-23

**Authors:** Liliana J. G. Silva, André M. P. T. Pereira, Angelina Pena, Celeste M. Lino

**Affiliations:** LAQV, REQUIMTE, Laboratory of Bromatology and Pharmacognosy, Faculty of Pharmacy, University of Coimbra, Polo III, Azinhaga de Sta Comba, 3000-548 Coimbra, Portugal; amptpereira@ff.uc.pt (A.M.P.T.P.); apena@ci.uc.pt (A.P.); cmlino@ci.uc.pt (C.M.L.)

**Keywords:** citrinin, foods, supplements, occurrence, analytical methods

## Abstract

Citrinin (CIT) deserves attention due to its known toxic effects in mammalian species and its widespread occurrence in food commodities, often along with ochratoxin A, another nephrotoxic mycotoxin. Human exposure, a key element in assessing risk related to food contaminants, depends upon mycotoxin contamination levels in food and on food consumption. Commercial supplements, commonly designated as red rice, usually used in daily diets in Asiatic countries due to their medicinal properties, may pose a health problem as a result of high CIT levels. In addition to the worldwide occurrence of CIT in foods and supplements, a wide range of several analytical and detection techniques with high sensitivity, used for evaluation of CIT, are reviewed and discussed in this manuscript. This review addresses the scientific literature regarding the presence of CIT in foods of either vegetable or animal origin, as well as in supplements. On what concerns analytical methodologies, sample extraction methods, such as shaking extraction and ultrasonic assisted extraction (UAE), clean-up methods, such as liquid-liquid extraction (LLE), solid phase extraction (SPE) and Quick, Easy, Cheap, Effective, Rugged and Safe (QuECHERS), and detection and quantification methods, such as thin layer chromatography (TLC), high performance liquid chromatography (HPLC), capillary electrophoresis (CE), biosensors, and ELISA, are also reviewed.

## 1. Introduction

Mycotoxins, toxic secondary metabolites, are produced by some fungal species, which readily colonize crops, contaminating them in the field or after harvest, and processed foods under certain favourable conditions of moisture, water activity, and temperature. They co-contaminate an array of agricultural products (e.g., cereals, legumes, nuts, oilseeds and spices) and their complementary goods worldwide [1].

More than 400 mycotoxins have been identified and reported. The most common toxins that attract notable attention in the contaminated agro-food products are aflatoxins (AFs), ochratoxin A (OTA), trichothecenes (deoxynivalenol (DON) and nivalenol (NIV)), fumonisins (FBs), zearalenone (ZEN), citrinin (CIT) and patulin (PAT) [1,2]. They are produced by some species of toxigenic fungi such as *Aspergillus*, *Penicillium*, *Fusarium*, *Alternaria* and *Monascus* [3,4].

CIT was first isolated by Hetherington and Raistrick from a culture of *Penicillium citrinum* Thom, prior to World War II in 1931. Later, it was identified in more than a dozen species of *Penicillium* (*P. citrinum*, *P. expansum*, *P. radicicola*, *P. verrucosum*), as well as certain strains of *Penicillium camemberti* (used to produce cheese), and numerous species of *Aspergillus* (e.g., *Aspergillus terreus* and *Aspergillus niveus*), including *Aspergillus oryzae* used to produce sake, miso, and soy sauce. CIT has also been isolated from *Monascus ruber* and *Monascus purpureus*, industrial species used to produce pigments [5,6]. These strains are traditionally used in China to produce red and yellow pigments for food [7]. These *Monascus* species have been used in food production and preservation in the Orient for centuries. Traditional applications included red wine brewing, red soybean cheese processing, food colouring and meat preservation. In addition, *Monascus* products have been used in medicinal therapy, being prescribed in several circumstances [8].

In an experiment in 1987 nearly 1400 *Penicillium* isolates were collected from several cultures, isolated directly from food and feed, and it was concluded that CIT was produced by the three species above mentioned [9]. *P. citrinum* is a mesophile growing in the temperature range of 5 to 40 °C, with an optimum between 26 and 30 °C. It grows over the pH range of 2 to 10, with an optimum pH between 5.0 to 7.0. It is a xerophile, with a minimum aw for growth between 0.8 and 0.84 [10]. It is the main mycotoxigenic fungal species described in rice (25–30 °C, humidity 30–35%) [11]. CIT is produced at temperatures ranging from 15 to 37°C with an optimum at 30 °C, but no information exists regarding the effect of aw on toxin production [10].

CIT is rapidly absorbed and distributed, namely to the liver and kidney [12]. A recent CIT toxicokinetic study in humans showed that 40% of CIT was excreted in urine [13], so its absorption was ≥40% [12].

CIT, although discovered due to its antibiotic properties against Gram-positive bacteria, has never been used as a drug due to its high mammalian nephrotoxicity [7,14]. The kidney is the major target organ of CIT toxicity, but other target organs such as liver and bone marrow have also been reported [7]. CIT is connected to yellow rice disease in Japan and it is a potent nephrotoxin in animals [9]. It has been implicated in several disease outbreaks in animals and humans [15]. Its acute toxicity varies with different species [9]. The 50% lethal dose is 57 mg/kg, 95 mg/kg, and 134 mg/kg for ducks, for chickens, and for rabbits, respectively [5]. It affects monogastric domestic animals such as pigs and dogs [10]. CIT results in necrosis of the distal tubule epithelium in the kidneys, alters the function, and degenerates the processes of the renal tubules [12]. CIT is a known hepatonephrotoxin, which causes functional and structural kidney damage as well as alterations in liver metabolism. It inhibits several enzymes linked to the respiratory chain of the kidney cortex and the liver mitochondria [16].

The effects of CIT can synergize with other mycotoxins, namely OTA and PAT, to inflict more pernicious effects on tissues and organs [17]. It can act synergistically with OTA to depress RNA synthesis in murine kidneys; however, it appears to be considerably less toxic than OTA [5]. CIT causes necrotic changes of parenchyma organs [18] and also increases the toxicity of OTA, whether additively, synergistically, or antagonistically, causing nephrotoxicity, gastrointestinal ailments, fetal malformations, and lymphoid tissue damage [7,19]. Other additive and synergistic interactions have occurred together with fumonisin B1 (FB1) and OTA, manifesting in cytotoxicity in human peripheral blood mononuclear cells [19].

CIT and OTA can trigger porcine nephropathy and have been implicated in the etiology of Balkan endemic nephropathy (BEN) in humans. It is implicated that CIT acts synergistically with OTA to cause BEN in humans, CIT being a clearly less potent nephrotoxin than OTA. Co-exposure to CIT and OTA has resulted in the modification of DNA adduct formation with development of C-C8dG-OTA DNA adduct [6,19]. In humans, CIT and OTA have also been reported to be causative agents of hepatic and renal carcinogenesis [19]. Nonetheless, CIT is classified by IARC in group 3 because of its non-ability to be carcinogenic to humans, and because of limited evidence in animals [20].

The studies undertaken in Bulgaria, Croatia, and Serbia addressed mixtures involving OTA, CIT, and FB1 due to their possible involvement in endemic nephropathy (EN). Higher co-contaminations with OTA and CIT or OTA and FB1 were found in EN villages than in non-EN villages. These studies confirmed that EN populations were more frequently exposed to OTA and CIT due to microclimatic conditions, such as high humidity, and specific dietary habits [21].

Citrinin might even be a more common contaminant all over the world since it can be synthesized by the same fungus which produces the globally found mycotoxin OTA [22].

The descriptions in the scientific literature reveal the presence of CIT in foods of either vegetable or animal origin, whether natural or resulting from fermentative processes. In the first, it is generally formed after harvest and occurs mainly in stored grains, being most relevantly in cereals and derivatives, though others, such as olives, apples, spices, fruit and vegetable juices, and beers, may also be contaminated, with lower contents [23,24,25,26]. With regard to foods of animal origin, most relevant are cheese [27], infant formulas, or dry meat products such as fermented sausages [5]. CIT is also found in red yeast rice, widely used in Asia as a food additive or in the production of wine [22].

In addition to CIT′s worldwide occurrence in foods of either vegetable or animal origin and in supplements, this manuscript presents a review of extraction methods, such as shaking extraction and ultrasonic assisted extraction (UAE), clean-up procedures, such as liquid-liquid extraction (LLE), solid phase extraction (SPE), Quick, Easy, Cheap, Effective, Rugged and Safe (QuEChERS), and detection and quantification procedures, such as thin layer chromatography (TLC), high performance liquid chromatography (HPLC), and capillary electrophoresis (CE), and others such as biosensor-based techniques and still enzyme-linked immunoassays (ELISA).

## 2. Physicochemical Properties

**Figure 1:**
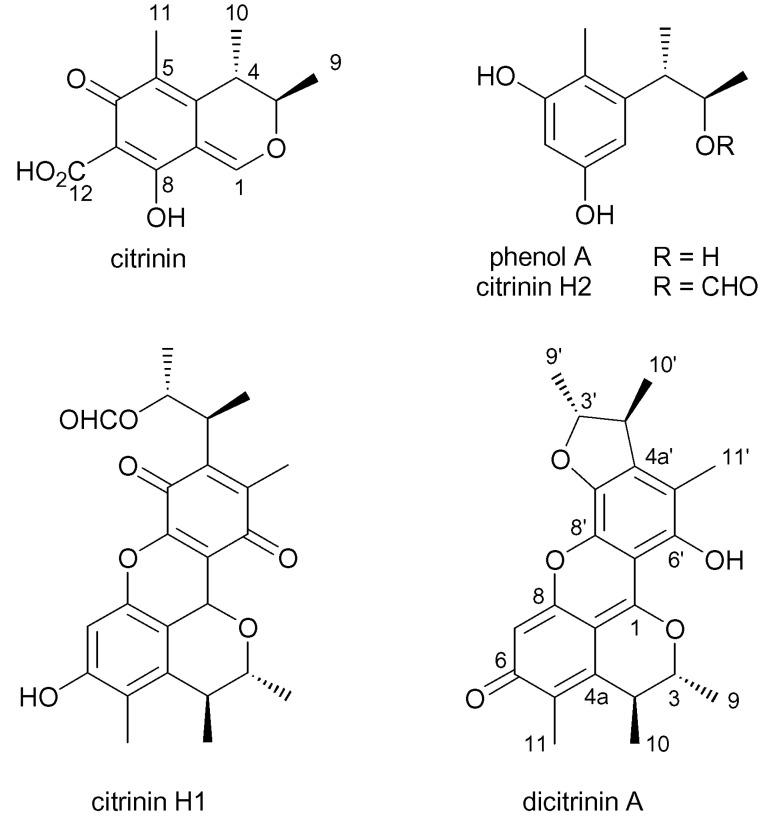
Chemical structures of citrinin and its decomposition products citrinin H1, citrinin H2, dicitrinin A and phenol A (based on Clark et al. [31]).

CIT (Figure 1) is a quinone methide with two intramolecular hydrogen bonds (Doughari, 2015). Its IUPAC Name is (3R,4S)-8-hydroxy-3,4,5-trimethyl-6-oxo-4,6-dihydro-3*H*-isochromene-7-carboxylic acid, the chemical formula is C_13_H_14_O_5_ (CAS Number: 518-75-2), and the molecular weight 250.25 g/mol [28].

Citrinin is an acidic lemon-yellow crystalline substance with maximal UV absorption at 250 nm and 333 nm in methanol solution. Its solution changes color, from lemon-yellow at pH 4.6 to cherry red at pH 9.9. It is sparingly soluble in water but soluble in dilute sodium hydroxide, sodium carbonate, or sodium acetate, and in methanol, acetonitrile, ethanol, chloroform, acetone, ethyl acetate, and most of other polar organic solvents [23,29,30]. It has a melting point of 172 °C/178.5 °C and can form chelate complexes and be degraded in acidic or alkaline solution or by heating [29,30]. Its pKa and log P values are 2.3 and 1.23, respectively [16].

## 3. Degradation Products

Degradation of CIT depends on the temperature and humidity conditions. Decomposition occurs at >100 °C in the presence of water, and >175 °C under dry conditions. Known decomposition products include citrinin H2 (CIT H2) (Figure 1), which shows much weaker cytotoxicity to HeLa cells than CIT and citrinin H1 (CIT H1), which is made up of two citrinin molecules, at 100 °C for 30 min or at temperatures above 100°C, and shows an increase in cytotoxicity as compared to the parent CIT. In 2006, another decomposition product was also reported, the cytotoxic citrinin dimer, dicitrinin A, together with other monomeric and dimeric degradation products [30,31,32]. It was found that after boiling in water, concentration of citrinin in *Monascus* dramatically decreased; 20 min of heating could decrease the concentration of CIT by 50%. These facts indicate that CIT is unstable and thermolabile in aqueous solution [30]. So, due to heat sensitivity CIT, is considered unstable, and its presence in processed foods is probably at lower levels.

## 4. Occurrence in Foods

The last report of the European Commission (2018) showed that about 96% of notifications of mycotoxins in the Rapid Alert System for Food and Feed (RASFF) concerned food, and notifications of citrinin are below 0.5% [33]. Table 1 presents the worldwide incidence and occurrence of CIT in different foods and supplements.

### 4.1. Cereals and Derivatives

CIT occurrence in food has been described worldwide, in Europe, Asia, North America, and Africa, as shown in Table 1. Usually, cereals are the most reported contaminated foods, rice being one of the most often evaluated. In addition, wheat and maize have also been mentioned as containing CIT.

#### 4.1.1. Rice

Rice cultivation is carried out in subtropical environments with sufficient warmth and high humidity levels (35–50%), resulting in invasion by CIT producing fungal spores in the field, during harvest and storage [36,55].

CIT has been found as a natural mycotoxin contaminant of rice grains. This cereal is essential for human diet and the main nutritional source for a third of the world’s population [11], being a very important foodstuff for billions of people. It is the dominant grain for half of the world’s population and provides 20% of the world’s dietary energy supply, being the major staple food in Asia, whereas wheat and maize supply 19% and 5%, respectively [36,55].

The natural occurrence of CIT in rice has been described in different countries [11], mainly in Asia [36]. As shown in Table 1, CIT was detected from the central region of Vietnam [34], Japan [11], and India [36]. In the first, 13% of the analysed samples (*n =* 100) were contaminated with levels between the limit of detection (LOD) (0.11 µg/kg) and 0.42 µg/kg, with mean levels of 0.38 µg/kg [34]. In Japan, 13.3% of the samples contained CIT ranging from 49 to 92 µg/kg [11]. In India, rice samples exhibited a frequency of detection and range identical to those found in Japan. In parboiled rice, 33.3% of the samples presented CIT in a range of 12 to 55 µg/kg [36]. The highest CIT levels were found in two samples from Canada, 700 and 1130 µg/kg [11]. On the contrary, in Spain, in 21 rice samples CIT was not detected (LOD < 1.5 μg/kg) [35].

Red rice, so-called red fermented rice (RFR), red mold rice (RMR) or red yeast rice (RYR), is traditionally prepared by fermenting normal rice grain with a fungal starter from the genus *Monascus*, notably *M. purpureus*, *M. pilosus*, or *M. ruber* [39]. This type of rice has been a mainstay in traditional Chinese medicine for thousands of years and, based on its medicinal properties, has been included by the Chinese in their daily diet as commercial food supplement, defined by The European Food Safety Authority (EFSA) as concentrated sources of nutrients or other substances with a nutritional or physiological effect that are marketed in “dose” form [16,38,39,56]. Its anti-hypertensive properties [39] and ability to reduce blood-lipid levels in humans, such as lowering total cholesterol, low-density lipoprotein (LDL), and triglycerides in the plasma of hyperlipidemic patients [16] are known, being appropriate in the primary and secondary prevention of heart disease and other complications of atherosclerosis, due to its main component, the monacolin K. Anti-diabetic [39,57] and antioxidant properties [35] were also reported.

Although RYR and products may be beneficial to health, reports exist of some *Monascus* species, principally *M. purpureus*, producing CIT during fermentation. Thus, contamination of rice fermented products with CIT has attracted attention and is a public health concern. CIT discovery in *M. purpureus* fermented red rice has cause much controversy about the safety of red mold rice products because up to 80% may contain this mycotoxin [38].

CIT was responsible for the so-called “moldy rice poisoning” case that occurred in Japan in 1953–54 [23]. Once the natural occurrence of CIT exists in widely-consumed-traditional RFR, to ensure safety it is important to accurately determine it in the RFR as well as in its related products [58].

*M. purpureus* YY-1 is widely used in food colorant production in China. Its pigments have been used as natural food colorants for over 1000 years worldwide, especially in China [59]. Recently, an increasing number of investigations have shown that *Monascus* pigments exhibit biological activities, such as anti-inflammatory, anticancer on colon cancer cells, and antihyperlipidemic activities [59,60]. RFR had also been long used as a natural food colorant in East Asia, and recently had also been used as a food additive and a dietary supplement in Europe and the U.S. [58].

CIT is a frequent contaminant in RFR products, and the contamination levels are often higher than those of other mycotoxins [37]. Contamination of RFR with this secondary metabolite has been reported in different studies in China [2,37,40,41,42,48,58], Croatia [16,38], Malaysia [39], Vietnam [11,34], and Taiwan [42].

In RFR from China, a level of 2903 μg/kg was detected [48]. The occurrence of CIT contamination in 12 RFR products collected from the major production areas of China was also studied, and 10 samples were found contaminated with high levels ranging between 140 and 44,240 µg/kg [37]. Moreover, traditional Chinese food red yeast rice, medicinal plants and their related products, accepted as functional foods or drugs or as dietary supplements worldwide, were analysed in order to evaluate the natural occurrence of CIT, whose presence has already become a threat to human health. Out of a total of 109 widely consumed samples analysed, CIT was detected in 31 (28%) ranging from 16.6 to 5253 μg/kg, all of them derived from 59 RYR and related products. None of the 50 medicinal plant samples analysed showed the presence of CIT [40].

In red rice samples from Malaysia (*n =* 50), the highest amount of CIT was at 20,650 μg/kg and lowest at 230 μg/kg, with a mean level of 4030 ± 4620 μg/kg [39].

In Croatia, six dietary supplements with RFR were analysed, two of which were contaminated with 95 and 98 µg/kg. In two grains of RFR, CIT was not detected [38]. In the same country, seven different commercially available cholesterol lowering products containing red yeast rice extract presented a frequency of detection of 28.6%, with levels oscillating between not detected (nd) and 98 µg/kg [16]. A level as high as 27,000 µg/kg was found in Taiwan [42].

#### 4.1.2. Maize

As concerns maize, among the 26 samples evaluated from Burkina Faso and Mozambique, CIT was quantified in three samples (12%), with a median of 1780 μg/kg and range between 531 and 5074 µg/kg [45].

CIT and dihydro-citrinone (DH-CIT) levels were evaluated in 204 maize samples harvested in Serbia in maize growing seasons with extreme drought (year 2012), extreme precipitation and flood (year 2014) and moderate drought conditions (years 2013 and 2015). The highest mean levels were observed in 2015 for both compounds, with 950 ± 2872 µg/kg, and 18 ± 33 µg/kg, respectively. CIT frequency was at 23% ranging from 7 to 10 058 µg/kg [46].

#### 4.1.3. Wheat

In Tunisia, a Mediterranean country characterized by warm temperature and prolonged wetness which promotes fungal proliferation and mycotoxins production, 200 samples of wheat were collected during 2010 and analyzed for CIT contamination. The results showed that its incidence was 50%, with contamination levels ranging between 0.1 and 170 μg/kg, in an average of 28 μg/kg [43].

CIT was also evaluated in wheat samples (*n =* 37) from the Canadian Great Lakes Region between 2011 and 2014, and, accordingly, its levels oscillated between <0.6 and 175.2 μg/kg [44].

In dusts of stored wheat grains from a loamy region in central Belgium, CIT was found at higher levels of between 137.0 and 343.9 µg/kg [61].

Cereals [47] and cereal products [48] were also evaluated in Croatia and Germany, respectively. The levels found in wheat and maize from Croatia varied according to the studied areas. In the Vukovar-Srijem region, the levels ranged between <1 and 103 μg/kg, with a mean level of 14.6 μg/kg, and in Osijek-Baranja area the range was <1–52.4 μg/kg, and the mean level 19.63 μg/kg [47]. In Germany, 61.1% of the samples, including wheat samples, were contaminated, with levels ranging between <1–2.7 μg/kg [48].

#### 4.1.4. Derivatives

In Nigeria, CIT and its metabolite, DH-CIT, were found in at least 61% and 4%, respectively, of the cereal-based food samples (family cereal, ogi and Tom bran) although as many as 53% of Tom bran samples contained DH-CIT. Only one sample of infant formula with milk and maize as constituents contained CIT [1]. It was found that the mean concentrations of CIT and DH-CIT were significantly higher (*p* < 0.05) in household-formulated complementary foods than in the industrially-processed foods [50]. In this country, the calculated range of margin of exposure (MOE) for CIT was 0–100 for chronic exposures of 0.002–102 μg/kg bw per day compared to the level of 0.2 μg/kg bw per day at which no concern for nephrotoxicity exists [1]. Later, studies showed that the percentage of the infants and young children (IYC) population (*n =* 110) at risk of adverse effects from dietary CIT exposures through complementary foods consumption was 19% [50]. One should note that in some countries, such as Nigeria, complementary foods are typically included in the diet of IYC when breast milk is no longer enough to meet nutritional needs. Cereals and nuts together with milk and their products are the major components of complementary foods for IYC. Consequently, they may be exposed to contaminated diets at the weaning stage, being the most vulnerable population in terms of mycotoxin exposure due to their young age, high intake of food and water per kilogram of body weight, fairly restricted diet, rapid rate of metabolism and growth, and a lower detoxification capacity [1].

In France, between 45 samples of breakfast cereals, 18%, were contaminated with CIT in the range of 1.5 to 42 µg/kg [49].

### 4.2. Other Foods

Beyond cereals, CIT was also found in other foodstuffs of vegetable origin (apples, black olives, nuts, spices), and also in foodstuffs of animal origin (e.g., cheese, cured meat) [6].

#### 4.2.1. Olives

Olives are grown mainly in southern European countries, such as Spain, Italy, Greece, and in Turkey. They are used to produce olive oil or to be directly consumed. Fermented olives are an important product worldwide [52]. During conventional olive production, the surface of the brine may be covered with a thick layer of mold. Despite the mold growth presenting the advantage of causing softening of the olive tissue, besides communicating a moldy taste and appearance, reducing the acceptable quality of olives and their shelf life, it produces mycotoxins. Some fungus, *P. citrinum* and *P. crustosum*, have been isolated from the surface of olives during fermentation [52]. During drying and storage of olives, *Aspergillus* and/or *Penicillium* strains are also able to develop on olive and produce OTA and/or CIT and/or aflatoxins type B [62]. In Turkey, CIT was detected in 20 of the 27 (74%) black olive samples from the Aegean Region, with levels oscillating between ND to 100 µg/kg, while in Marmara Region 81% were contaminated in a range of 75 to 350 µg/kg [52]. In black olive samples (*n =* 10) purchased from supermarkets and retail markets in Morocco, the amount of CIT found in 80% of the samples was between >LOD (0.2 µg/kg) and <LOQ (0.5 µg/kg) [51].

#### 4.2.2. Apples

A total of 351 apples of seven varieties were analyzed in Portugal including 14 samples (3.9%) contaminated with CIT, with mean levels comprised between 320 ± 680 µg/kg, for Richard variety, and 920 ± 630 µg/kg for Rome Beauty [24].

#### 4.2.3. Beer

Forty-nine samples of lager beers from Czech Republic were analysed for CIT and the results showed that the number of contaminated samples was very low. In this survey, only three beers were positive. CIT was not detected in 92% of the samples owing to its low stability and degradation during the brewing process [26]. Accordingly, there are few studies reporting CIT contamination of this beverage probably because CIT is destroyed during the mashing and wort boiling process. However, there was some reported contamination at trace levels, showing that insignificant levels of CIT remained after the short-term brewing process. According to Xu et al., after boiling in water, the concentration decreased by about 50% after 20 min of heating, indicating its instability and thermolability in aqueous solution [26,30].

#### 4.2.4. Spices

Regarding spices, Jeswal and Kumar, in 2015, analysed 311 samples of different spices, including red chili, black pepper, turmeric, coriander, cumin, fennel, caraway, fenugreek, and dry ginger, collected from local markets in rural and urban areas of the district of Bihar, India. Red chili samples showed the highest detection frequency, 47.2%, while dry ginger samples presented the highest mean concentrations, 85.1 µg/kg [54].

#### 4.2.5. Sufu, Cooked Foods, Ham, and Snacks

In China, sufu (*n =* 12), cooked foods (*n =* 15), ham (*n =* 23) and snacks (*n =* 7) samples were studied for content of CIT. The results showed positive in 40.4% of the analysed samples, with sufu samples presenting the highest frequency, 91.7%, and ham samples the highest mean concentration, 190 µg/kg [29].

#### 4.2.6. Cheese

Cheese is contaminated by CIT, where CIT-producing toxigenic strains directly grow in the cheese mass [6].

Cheeses, very sensitive products, are interesting foods which can be contaminated both naturally and artificially by CIT-producing fungi. Different domestic strains of *Penicillium* are used to produce cheeses, such as Camembert-type, French cheeses and various goat’s milk cheeses. *P. roqueforti* and *P. camemberti*, the most commonly used and studied species, do not appear to be able to produce CIT in cheese, however, accidental contamination of cheese by a wide variety of other wild strains of *Penicillium* can happen. On the other hand, *P. citrinum* and *P. expansum* do not produce CIT at 4 °C but do so at 20 °C, being able to produce quantities of up to 600 mg/kg after 10 days of incubation. Although contamination is mainly superficial, 33% of the toxin remains in cheese after trimming. Bailly et al. also verified that, for the evaluated cheeses, 50% of CIT still remains after eight days of storage [27].

By adding CIT to cheeses, the quantity decreased with storage time depending on the type of cheese. A slight decrease (10%) was observed after 6 h of storage for some cheeses (fresh goat, Saint Marcellin, and Soignon). Despite the gradual decrease with increase of storage time, >70% of the CIT was recoverable after eight days at 20 °C. For other cheeses (Roquefort, Raclette, Fourme, and Rouy), there was a rapid decrease after 6 h of storage, >33% at 20 °C, and about 50% of the amount added remained at the end of the assay (192 h). Another group of cheeses (Cantal, Reblochon, and Emmental) presented intermediate stability when compared to the other two groups. The storage temperature appeared to have only a tiny effect on the quantity of CIT recovered in the three types of cheese. This study showed that the stability of CIT in cheese is influenced neither by the temperature of storage nor by prior sterilization of the cheese. So this shows that its loss is a consequence of a chemical reaction with the cheese components, more than of a microbial action. Probably due to the high casein content in cheeses, the reactive groups of this protein interact with CIT causing its disappearance. Differences in pH may explain the differences in stability, as fresh goat’s cheese has a pH of 4.2 and Roquefort cheese has a pH close to 6 [27].

#### 4.2.7. Cured Meat

With regard to meat products, mycotoxins that assume greater importance from a public health perspective are aflatoxin B1 (AFB1), OTA, sterigmatocystin (STC), cyclopiazonic acid (CPA) and CIT. During the ripening period, the surface of dried traditional meat products is covered by fungi of *Penicillium* spp., *Aspergillus* spp. and *Eurotium* spp. whose spores mainly come from the environment in which the ripening chambers are located [18]. *Penicillium expansum* isolated from meat and apples produced both patulin and CIT [63].

Relatively high amounts of CIT were found on dry meat after a 16-day incubation period with *P. citrinum*, at 20°C (87 mg/kg) [64]. CIT production by *P. citrinum* on meat samples was rapidly observed after four days. Levels, as high as 86.9 mg/kg, were obtained after 16 days of culture on dry cured ham. More than 50% of the CIT initial content was lost after only 6 h of incubation at 20 °C, while after 192 h of incubation less than 15% of the toxin remained [64]. The study of CIT stability at 20°C and 4°C demonstrates that the half-life of the toxin is about 6 h, suggesting that it is only partially stable on dry cured ham. This result agrees with those obtained on some cheeses [64].

Few data on CIT content in dry-cured meat products are found across the literature despite CIT-producing fungal strains having been isolated from dry-cured meat products [53]. Markov et al. showed that CIT was not a significant meat products’ contaminant. The fermented dry meat products (*n =* 50) showed a low frequency, 6%, and levels between <1.0–1.3 µg/kg, while semi-dry sausages (*n =* 25) presented levels <1.0 µg/kg in 4% of the samples [53].

There is an important need to carry out control on different meat products due to climatic variations over the years of production of cured meat, since, as for any mycotoxin, the CIT content varies. Another factor that can facilitate the diffusion of mycotoxins from the surface into dry fermented products is the damage caused to the outer coating. This fact has already been proven in the OTA entry from the surface into products based on cured meat during long-term ripening [65] and AFB1 during the ripening of dry-fermented meat sausages [66].

### 4.3. Legislation

In the EU, the Regulation (EU) No. 212/2014, concerning the maximum allowed presence of CIT in food supplements based on rice fermented with red yeast *M. purpureus* established a maximum value of 2000 μg/kg [67]. China and Japan have set a maximum limit of 50 and 200 μg/kg, respectively, for CIT in fermented red rice [68]. However, the acceptable levels of this mycotoxin in other food and feed commodities have not yet been regulated in different areas of the world, including Europe.

## 5. Analytical Methods

Since the stability of CIT is affected by different factors, such as temperature, solvent composition used for sample extraction and HPLC mobile phase, the need to use a sensitive methodology becomes evident for its determination in various foodstuffs [30]. Table 2 presents the analytical methodologies for determination of CIT in foods and supplements reported in the scientific literature.

### 5.1. Extraction

Extraction methods are key factors that influence recovery rates. Commonly, acetonitrile (ACN) and methanol (MeOH) were the most selected extraction solvents for CIT determination in foods (Table 2).

ACN has usually been used in different mixtures: ACN-4% aqueous solution of NaCl (9:1, *v*/*v*) with pH adjusted at 1.5 with undiluted HCl [34], ACN-5% formic acid (FAc) [35] in rice analysis; ACN/H_2_O/acetic acid (79:20:1, *v*/*v*/*v*) used in maize extraction [45,46], wheat [44], and complementary foods for infants and young children [1]. 4% aqueous solution of KCl acidified to pH 1.5 with undiluted H_2_SO_4_ and ACN were used in cereals [49] and in olives [51]; ACN (180 mL)/4% KCl (20 mL)/20% H_2_SO_4_ (2 mL) in olives [52]. ACN-4% aqueous KCl (9:1) [24] [74]; ACN-KCl (5%; 80:20, *v*/*v*) acidified by H_2_SO_4_ to pH 3 were applied to cheese [27] while ACN:4% KCl (9:1, *v*/*v*) acidified to pH 3 with H_2_SO_4_ was used in dry cured ham [64]. Finally, ACN containing 100 mM citric acid was used in fruits’ analysis [75] (Table 2).

The mixture MeOH:H_2_O has been widely used in different proportions. It has been used in RYR and related products [40], rice [68], spices [54], and fermented meat products [53] at 70:30 (*v*/*v*). In various cereals, maize, wheat and rice, it was used in a proportion of 2:8 (*v*/*v*) [71], and in grains of red fermented rice, tablets and capsules a proportion of 80:20 was used [38]. Methanol was applied alone in RFR [37].

Other solvent mixtures, such ethanol: H_2_O (E:W 7:3, *v*/*v*) was used in RFR by means of ultrasonic (US) and shaking extraction [58]. Besides these techniques, other extraction methods were also attempted: US extraction with TEF solvent mixtures (toluene:ethyl acetate:formic acid, 7:3:1, *v*/*v*/*v*); shaking extraction with EW; shaking extraction with EF solvent mixtures (ethyl acetate:formic acid, 1:1, *v*/*v*); shaking combined with US extraction in EW. It was demonstrated that shaking combined with ultrasonic extraction in EW was the most efficient extraction method. However, this procedure is very time consuming since the extractive process takes 2.5 h. Among several solvents to extract CIT from RMR, E:W (75:25%), at 80 °C, followed by shaking during 30 min, presented the best results [42].

Ultrasonic assisted extraction was employed in sufu, cooked foods, ham, and snacks [29].

Dichloromethane (DCM) with 0.5 M phosphoric acid was also used for CIT extraction of cereals and cereal products [48]. The mixture toluene-ethyl acetate-FAc (7:3:1, *v*/*v*/*v*) was occasionally used for extraction as, for example, in Xuezhikang capsules and other *Monascus*-fermented products [41].

As mentioned above, some extraction procedures promote acidification. This is done in order to improve recovery and reproducibility. Best results were obtained for pH 1.5 (80.3 ± 5%) than pH 4 (23 ± 1.5%) [49]. Acidification is made using undiluted HCl [34], undiluted H_2_SO_4_ [27,49,51,64], or H_3_PO_4_ [48].

### 5.2. Clean-Up

In some procedures, after extraction only centrifugation followed by filtration is used for clean-up. Centrifugation is usually performed at 3000 rpm/5 min [58], 3000 rpm/2 min [46] or 10 min [16], 3000 g/10 min [38], 6793 g/10 min [37]. Filtration through a 0.45 µm pore size filter is usual [16,38,58], however, 0.22 µm filter is also applied [37]. In some procedures, only sedimentation by gravity is used [1].

#### 5.2.1. Liquid-Liquid Extraction (LLE)

Liquid-liquid extraction (LLE) is based on the different solubilities of the toxin and nonpolar contaminants, the former being usually soluble in the aqueous phase and the others in the immiscible organic phase. To remove lipids and cholesterol, n-hexane is usually used. However, this procedure is dependent on the matrix where it is being used, and which compounds are being determined. It is also time consuming and, due to adsorption on the glass material, sample loss may occur [76]. Some procedures use n-hexane for defatting. For example, the oil from some matrices such as black olives [51,52], breakfast cereals [49], and rice [34,43] was extracted with n-hexane preventing it from interfering with the assay.

Some procedures use liquid-liquid partition (LLP) for extract clean-up. This step, using chloroform (ClCH_3_), was applied to rice extracts [34,43], breakfast cereals [49], and black olives [51,52].

#### 5.2.2. QuEChERS

One fast and non-laborious method, Quick, Easy, Cheap, Effective, Rugged and Safe (QuEChERS), based on the extraction/partitioning process, was developed with H_2_O and ACN containing 5% Fac, avoiding the need for further rice extract clean-up. Afterwards, ultra-high-performance liquid chromatography (UHPLC) coupled with fluorescence detection was used for detection and quantification [35]. The used partitioning salts were MgSO_4_, NaCl, tri-sodium citrate dehydrate and sodium hydrogen citrate sesquihydrate (an extraction step based on partitioning via salting-out, involving the equilibrium between an aqueous and an organic layer) [35]. MgSO_4_ and NaCl salts with a citrate buffer improved the overall recoveries and the addition of water to the sample before extraction to hydrate and swell the rice matrix positively affected extraction efficiency [77].

#### 5.2.3. SPE

Nowadays SPE is by far the most popular technique used in routine analysis of mycotoxins. Immunoaffinity columns (IAC) have been applied to extracts of different matrices, such as RYR and related products [40], corn and wheat [47], and fermented meat products [53] for the determination of CIT.

The use of polyamide columns for clean-up of cereals and cereal products was highly applied to RFR, regarded as a difficult matrix because of the very high content of coloring agents [48].

Molecularly imprinted polymer (MIP) columns were introduced as an alternative to IAC, as they can be recycled in order to reduce costs [78]. One novel magnetic MIP (m-MIP) was synthesized for clean-up of rice sample extracts containing CIT prior to its determination by HPLC with UV-DAD [68]. The m-MIPs can be reused for sample analysis in at least 30 extraction cycles, without significant loss in performance or reproducibility [68]. Besides, the method is faster, avoiding the need of SPE column packing or filtration operations, and operationally simple thanks to the ease with which the magnetic particles can be removed. Therefore, it provides a promising choice for the determination of CIT in food matrices [68]. MIPs have been previously proposed for selective SPE of CIT in rice with recoveries in the range of 86.7–97.7% [79]. A disposable evanescent wave fiber optic sensor coated with a MIP (composed of a naphthylamide-based fluorescent monomer, which exhibits fluorescence enhancement upon binding with carboxyl-containing molecules) containing a fluorescent signaling group on a 4-cm long polystyrene optical waveguide was used for determination of CIT and 2,4-dichlorophenoxyacetic acid (2,4-D) [80].

In order to clean-up crude extracts of different fruits (apples, oranges, sweet cherries and tomatoes), a self-made SPE column containing aminopropyl (NH_2_) and mixed-mode cationic exchange (MCX) adsorbents was used for UHPLC-MS/MS determination [75]. This column offered an important advantage, a single-step clean-up instead of expensive IAC and MycoSep multi-functional clean-up column, which can significantly shorten the sample preparation time, with superior recoveries and minimum matrix effects, compared to the conventional method [75].

For beer samples, Lhotská et al. [26], after filtration with a 0.45 um filter, used direct injection of 100 μL filtered beer samples into an on-line SPE (fused-core Ascentis Express RP C18)-HPLC system. This procedure enabled fast and effective sample extraction including separation in less than 6 min.

Some researchers opted, after extraction, for methodologies based on **the** dilute and shoot (DaS) approach. Malachová et al. [73] proposed this, followed by LC-MS/MS for quantitative determination of 295 fungal and bacterial metabolites, including CIT, in four types of different food matrices, apple puree, hazelnuts, maize and green pepper. The repeatability of the method was acceptable (RSD ≤ 20) for 97% of all analytes in apple puree and hazelnuts, for 95% in maize and for 89% in green pepper. Previously, this method was used for the quantification of 87 analytes, in breadcrumbs and moldy food samples [69], there being a need to lower the pH from 4 to 1.5 in order to increase the extraction from 23% to 80%. Hajnal et al. [46], following the method recommended by Malachová et al. [73] and Kos et al. [72], after extraction with a mixture (ACN/water/acetic acid 79:20:1, *v*/*v*/*v*), shaking and centrifugation, also used DaS to evaluate different mycotoxins, including CIT, in maize harvested in Serbia during 2012–2015. The same methodology was employed in complementary foods for infants and young children consumed in Nigeria [1], and for maize [45]. The obtained results concluded that this approach is also possible in the case of complex matrices.

### 5.3. Detection and Quantification

Instrumental selection is one of the most important factors influencing the sensitivity of quantification [37]. Common methods for CIT analysis are thin-layer chromatography (TLC) and high-performance liquid chromatography (HPLC) with UV diode-array (UV-DAD), fluorescence detection (FD), and mass spectrometry (MS) detection. Ultra-performance liquid chromatography (UPLC) is also used. Enzyme immunoassays (EIA), capillary electrophoresis (CE) biosensor-based and voltammetry techniques are rarely or occasionally used.

#### 5.3.1. TLC

In the past, TLC was chosen for CIT determination. This technique was applied to apple purified extracts by Martins et al. [24], under UV light at 366 nm; also to black olives [52], cheese [27] and dry cured ham with fluorimetric detection at 330 nm [64].

#### 5.3.2. LC-FD and UPLC-FD

Due to poor sensitivity, TLC was replaced by HPLC-FD, with numerous advantages such as simplicity, high sensitivity [23,35] and adequate recoveries, due to the acidic nature of CIT molecule which exhibits native fluorescence [23]. According to Arévalo et al. [81], the weak fluorescence of CIT can be greatly enhanced in acidic media.

CIT has a conjugated, planar structure which provides its natural fluorescence (the highest fluorescence is produced by a non-ionized citrinin molecule at pH 2.5) [23]. Compared to UV detection, FD is about 100 times more sensitive, thus becoming the preferred choice for routine determination [30,35]. So CIT fluorescence detection has been intensively adopted considering that this technique shows a greater sensitivity than HPLC with UV-Vis detection. CIT has an absorption maximum at λ = 332 nm and an emission maximum at λ = 521 nm [81].

As depicted in Table 2, HPLC-FD has been successfully applied for the analysis of CIT in cereals, which includes rice and dietary supplements derived therefrom, derivatives, olives, beers, fermented meat products, sufu, ham or snacks. HPLC-FD has been used in extracts of rice [34], RFR and related products [37,38,40,41,42,58], wheat [43], corn and wheat [47], breakfast cereals [49], black olives [51], lager beers [26], fermented meat products [53], sufu, cooked foods, ham, and snacks [29].

The λ ecx vary between 330 nm and 350 nm and λ em between 495 and 500 nm (Table 2), despite that some authors consider that CIT has an absorption maximum at λ exc = 332 nm and an λ em maximum at 521 nm [81]. HPLC-with UV DAD at 331 nm was used for rice [68]. For beers, preconcentration of OTA and CIT from beer samples was performed on an Ascentis Express RP C18 guard column (5 × 4.6 mm), particle size 2.7 μm. Fluorescence λ exc/em were set at 335/497 nm. The choice of the λ exc/em was carried out as a compromise between the fluorescence spectra of OTA and CIT in mobile phase and sensitivity of determination for both mycotoxins [26].

As shown in Table 2, reverse phase columns, such as C18, were usually used in CIT detection and quantification. The column temperatures used are variable, usually between 25 °C [82] and 50 °C [83]. Regarding flow rates, the oscillations are comprised between 0.4 mL/min [48] and 1 mL/min, the most frequently used [29,37,38,40,41,42,43,53,58]. According to the chromatographic conditions, retention times for CIT oscillated between 1.25 [82] and 19 min [84]. When UPLC-FD was used, temperature and flow rate were at 45 °C and 0.5 mL/min, respectively [35].

Due to the acidic nature of CIT, and since its anion is not fluorescent, the mobile phase in reverse phase (usually C18 column) must be acidic in order to obtain a high analytical signal. This acidification is usually obtained using phosphoric acid (H_3_PO_4_) in different proportions, in mixture with ACN [47], H_2_O: ACN [37,40,41,53,58], or ACN: propan-2-ol [34,43,49,51]. The molarity of the H_3_PO_4_ influences the peak form, once the decrease in pH improves its form and the retention time increases [48].

ACN is also used in mixture with FAc [38], acetic acid [26], or trifluoroacetic acid (TFA) [42]. Acetic acid and FAc were added to water (solvent A) and ACN (solvent B) at different concentrations. The fluorescent signal of CIT increased with concentration, achieving a maximum and remaining constant from 1% acetic acid and 2% formic acid, the latter providing higher sensitivity [35]. Huiqin et al. [29] used only ACN:H_2_O (35:65).

Usually ACN, used as an organic eluting solvent in the mobile phase, is preferred over MeOH as CIT shows higher fluorescence in it [35] and, as in MeOH, the molecule is much more solvated [85].

However, some researchers use MeOH instead of ACN. MeOH has been used in mixture with ethyl acetate and H_3_PO_4_ [48], or with acetic acid [26]. Meister et al. [48] verified that the CIT peak form was influenced by the molarity of the phosphoric acid in the mobile phase as well as by the mixing proportion of the organic and aqueous components (55% methanol, 10% ethyl acetate). The peak form improved with pH decrease, and the CIT retention increased with increasing aqueous H_3_PO_4_ content [48].

When HPLC-FD is used, LODs, expressed in ng/g, vary between 0.11 ng/g for rice [34] and 2.8 ng/g for rye [48]. For supplements, LODs oscillated between 0.8 ng/g [40] and 1.0 ng/g [37], however when the results are presented in mg/mL the values were comprised, between 0.187 [41] and 0.5 [38]. The lowest values were obtained for lager beer, 0.006 ng/mL [26]. Regarding LOQs, the values oscillated between 0.35 ng/g [34] and 10.3 ng/g [48] for rice. Regarding supplements, the only value found for LOQ, expressed in ng/g, was 3 ng/g [37]. These values expressed in ng/mL were between 0.6 [41] and 1.0 [38]. Like LODs, beers also showed the lowest LOQs, 0.02 ng/mL [26] (Table 2).

UPLC-FD is an interesting alternative for determination of CIT, since it provides faster separations with better resolutions. So UPLC coupled to fluorescence detection was used for rice extracts and the obtained LOD and LOQ were 1.5 and 5.0 ng/g, respectively [35].

#### 5.3.3. LC-MS/MS and UPLC-MS/MS

In spite of the high specificity and sensitivity of FD, there is an increased interest in MS detection, once it allows an unambiguous analyte identification, regardless of its high cost and matrix effects such as ion suppression or enhancement phenomena [85]. So alternative LC systems have been proposed such as LC-MS/MS, for complementary foods for infants and young children [1], maize extracts [45,46], RFR [37,40], wheat [44], and spices [54]. UPLC-MS/MS determination was used for extracts of fruits (apples, oranges, sweet cherries, and tomatoes) [75].

The column temperatures used are variable, usually between 20 °C [82] and 30 °C [54]. The flow rates were between 0.25 mL/min and 1 mL/min [1,42,44,45,46].

Regarding the mobile phase composition for LC-MS/MS, inversely to what happens with LC-FD, MeOH seems to deserve the preference of researchers. For example, MeOH has been used in mixture with H_2_O, acetic acid, and ammonium acetate [1,45,46], with H_2_O, ammonium acetate, and FAc [54], ammonium acetate and Fac [54]. ACN has also been employed with FAc [40].

The majority of authors use multiple reaction monitoring (MRM) with electron spray ionization (ESI) in positive mode (+) when UPLC [75] or HPLC [37,40,54] were used. Some researchers did not specify the ESI mode [1,42,44,45,46].

The precursor ions selected were 251.0 (*m*/*z*) [1,37], 251.1 (*m*/*z*) [40,44,46,72,73] or 251.2 (*m*/*z*) [75]. The product ions were 205 (*m*/*z*) and 233 (*m*/*z*) [37], 233.0 *m*/*z* and 205.1 *m*/*z* [40], 191.0/205.2/233.1 [44], 233.2/205.21 [46], 233.0/205.2 [1].

The LODs values varied between 0.16 ng/g for maize [46], and 250 ng/g also for maize [45]. The LOQ values were between 1.4 ng/g for wheat [44] and 3.0 ng/g for RFR [37].

The matrix effect can be compensated using different quantification strategies such as matrix-matched calibration or external calibration using isotopically labelled internal standards [85].

#### 5.3.4. Immunoassays

Enzyme-linked immunoassays (ELISA), indirect competitive-ELISA (ic-ELISA), and enzyme immunoassay (EIA) have been employed in spices [54], cereals such as maize, wheat, and rice [48,71], and cereal products [48].

ELISA is a sensitive and high-throughput method usually used for sample screening and quantification due to its low cost. However, due to cross-reactivity, false negatives are generated, which require confirmation by LC-MS/MS [54] or LC-FD [48]. Kong et al. [71] prepared a monoclonal antibody (mAb)1F2 and developed an indirect competitive ELISA (ic-ELISA) and a lateral-flow immunochromatographic assay (ICA) strip for the detection of CIT in maize, wheat, and rice. Li et al. [86] developed a microsphere-based flow cytometric immunoassay for the determination of CIT in RYR.

On rare occasions mycotoxin determination employs capillary electrophoresis (CE) and biosensor-based techniques [18]. CE is a powerful analytical technique which is designed to separate the species, based on their charge to size ratio in an electric field in a small capillary. The main advantages of CE over the HPLC technique include the low sample volume required, the low solvent consumption of solvents and reagents, the environmental friendliness, cost efficiency, simplicity, high resolution and the short time analysis [16]. Nigović et al. [16] developed, for the first time, a simple micellar electrokinetic chromatography (MEKC) method to achieve simultaneous quantification of lovastatin in the red yeast rice existing in lactone and hydroxy acid forms, as well as CIT, as toxic fermentation by-product, compounds present in various commercial formulations of RYR [16].

Capillary zone electrophoresis (CZE) separation coupled to a UV detector (at 319 nm) was used as the determination method of CIT in red yeast powder, after clean-up by IAC, because of its simplicity, high speed, highly efficient separation and minimal solvent and reagent consumption. Adequate recoveries were obtained [87].

Novel sensors have also been developed, such as quartz crystal microbalance sensor with recoveries ranging from 85.8%, for rice, and 94.5%, for white rice vinegar, fortified at 10 and 100 μg/kg, respectively [88]. A molecular imprinted voltametric biosensor was applied to rye samples (recoveries between 96.30% and 101.35%) [89], and a molecular imprinted surface plasmon resonance (SPR) biosensor was used in RYR [90]. Electrochemistry offers the portability of a miniaturized sensor of CIT. Nasir and Pumera [91] showed that voltammetry on edge-plane pyrolytic graphite (EPPG) electrode offers excellent selectivity and sensitivity towards simultaneous detection of zearalenone and CIT. However, these costly methods require trained personnel, sophisticated instruments and complex sample preparation steps [71].

A fast, selective and very sensitive methodology based on an electrochemical immunosensor incorporated in a micro fluidic cell was developed for CIT in rice samples [81]. A microsphere-based flow cytometric immunoassay (MFCIA) [86] and a MIP-based biosensor were also reported for determination of CIT in RYR, with recoveries between 89% to 94%, and 97.96% to 101.28%, respectively [90].

## 6. Conclusions

From the different studies carried out on the occurrence of CIT in foods and supplements, a high variability in frequency and detected levels has been observed. These studies also reveal a wide dissemination in different continents, such as Europe, Asia, America, and Africa. Greater attention has been given to foods of vegetable origin, mainly cereals and derivatives. The data obtained so far show that the highest levels of CIT were found in maize in Serbia, with mean levels of 950 ± 2872 µg/kg [46], followed by *Tom bran* in Nigeria, with 160 ± 313.6 µg/kg [1]. Apples, analysed in Portugal, showed high levels, between 320 and 920 µg/kg [24]. CIT has also been evaluated in foods of animal origin, especially in ham and fermented meat products. While in the first levels these are of the order of 190 µg/kg [29], in the second matrix they are clearly lower, <1.0–1.3 µg/kg [53].

The lack of regulation regarding the CIT content in food creates a void that needs to be urgently filled, in order to avoid its presence in biological fluids with the inevitable harmful effects on human health.

With respect to supplements, usually they presented the highest levels of CIT. These values were 44,240 µg/kg [37], 20,650 µg/kg [39], 5253 µg/kg [40], 27,000 µg/kg and 13,550 µg/kg [42], which exceed the maximum limits regulated by the EU, Japan, and China.

Given the great diversity of matrices, several extraction and clean-up procedures have been proposed to obtain suitable extracts for the analytical instrumentation used in the detection and quantification of CIT. Among the first, different solvents have been used, with preference for ACN, used in mixture with aqueous solution of NaCl or KCl, whose pH is adjusted at 1.5 or 3.0, with undiluted HCl or H_2_SO_4_, or in mixture with water and acetic acid, with FAc, or with citric acid. As for the implemented clean-up procedures, centrifugation followed by filtration, liquid-liquid partition, QuEChERS, and solid phase extraction, the most popular procedure, with IAC, polyamide, MIP, and aminopropyl in mixture with MCX columns, have been employed.

More recently, “dilution and shoot” has been experienced. The DaS approach is a promising sample preparation method, especially when the concentration levels of target analyte are relatively high and the matrix components do not co-elute or interfere with ionization of the analyte of interest. However, this technique results in sensitivity sacrifice [92].

Until now, LC-FD has been the most commonly used analytical instrumentation to evaluate CIT in foods and supplements. However, lately LC-MS/MS has received more attention for direct quantification [1,45,46] or for confirmation [37,40,54]. As far as we know, UPLC-FD, HPLC with UV diode array, LC/DAD/FD/MS/MS, UPLC/ESI-MS/MS have been much less used.

Similarly to what occurs with other mycotoxins in foods [93] and medical herbs or traditional medicines [94,95,96] and with CIT in biological fluids, such as urine and plasma [13,20,84,97], the use of stable isotope labelled internal standards in the CIT analysis in complex food matrices is a very promising pathway to overcome the matrix effect.

## Figures and Tables

**Table 1:** Worldwide incidence and occurrence of CIT in foods and supplements.

Food/Supplement	Country	N° of Samples	Incidence (%)	Range (µg/kg)	Mean ± SD (µg/kg)	Median (µg/kg)	References
Rice	Japan	30	13.3	49–92			[11]
	Canada	2	100	700–1130			
Paddy rice	Egypt	30	33.3	4.36–20.36			[3]
Rice	Vietnam	100	13	LOD–0.42	0.38		[34]
White rice	Spain(Granada)	8	0	nd (<1.5)			[35]
Brown rice		8	0				
Red rice (supplement)		5	0				
Rice	Iran, 2010/11	65	26.6	5–21.05	10.45		[36]
Parboiled rice	India	18	33.3	12–55			
Rice	India	30	13	49–92			
Red fermented rice (RFR) (supplement)	China	12	83	140–44,240			[37]
Grains of RFR (supplement)	Croatia	2	0				[38]
Dietary supplement with RFR		6	33.3	95–98			
Red rice (supplement)	Malaysia	50	100	230–20,650	4030 ± 4620		[39]
Red yeast rice (RYR) (supplement)	China	59	28	16.6–5253			[40]
Red yeast rice (RYR) (supplement)	China	2	100	0.97–1.32			[41]
*Monascus* pigment powder		2	100	122–594			
Functional red yeast rice powder (supplement)		3	100	0.10–5.41			
Red yeast rice (RYR)-(supplement)	Croatia	7	28.6	nd–98			[16]
Red mold rice (RMR) (supplement)	Taiwan	2	100	5742–27,000			[42]
	China	3	66.6	49–13,550			
Wheat	Tunisia	200	50	0.1–170	28		[43]
Wheat	Canada	37	67.6	nd-175.2			[44]
Maize	Burkina Faso	26	12	531–5074		1784	[45]
	Mozambique						
Maize	Serbia	204	Citronone/Dihydrocitronone CIT/DH-CIT	CIT/DH-CIT	CIT/DH-CIT	CIT/DH-CIT	[46]
	2012	51	4/nd	10–48/nd	11 ± 3/nd	11/nd	
	2013	51	8/nd	5–547/nd	175 ± 2/nd	73/nd	
	2014	51	4/nd	2–6/nd	4 ± 3/nd	4/nd	
	2015	51	23/8	7–10058/2–68	950 ± 2872/18 ± 33	61/2	
Cereals	Croatia						[47]
	-Međimurje	20	-	<1			
	-Osijek-Baranja	15	80	<1–52.4	19.63	15.8	
	-Vukovar-Srijem	15	66.7	<1–103	14.6	1.23	
	-Brod-Posavina	5	-	<1–23.8			
Cereals and derivatives	Germany	18	61.1	<1–2.7			[48]
Breakfast cereals	France	45	18	1.5–42			[49]
Family Cereal	Nigeria	26	88.5	1.2–151	32.6 ± 39.8	25.1	[1]
Ogi		23	60.9	0.8–159	20.0 ± 40.8	7.0	
Tom bran		30	73.3	1.7–1173	160 ± 313.6	13.4	
Infant formula		17	5.9	3.6	3.6	3.6	
Household-formulated complementary foods (*Tom bran*)	Nigeria	53	67.9	0.8–1173	106 ± 25.4	9.5	[50]
Industrially-processed complementary foods	Nigeria	84	28.6	1.2–151	31.4 ± 39.4	21.9	
Black olives	Morocco	10	80	>LOD–<LOQ			[51]
Black olives	Turkey						[52]
	-Marmara	42	81	75–350			
	-Aegean	27	74	nd–100			
Apples	Portugal	351	3.9	320–920			[24]
Lager Beers	Czech Republic	49	8.2	<LOD–0.19 ug/L		<LOD	[26]
Fermented meat products	Croatia	90	5.55				[53]
game sausages		15	1/15 (6.66%)	<1.0–1.0			
semi-dry sausages		25	1/25 (4%)	<1.0			
fermented dry meat products		50	3/50 (6%)	<1.0–1.3			
Sufu	China	12	91.7	96–240	160		[29]
Cooked foods		15	6.67	110	110		
Ham		23	34.8	110–230	190		
Snacks		7	42.8	120	120		
TOTAL		57	40.4	96–240	160		
Spices	India						[54]
red chilli		55	26 (47.2%)		69 ± 12.5		
black pepper		42	19 (45.2%)		76.9 ± 17.8		
turmeric		35	0	0	0		
coriander		30	12 (40%)		81.0 ± 23.0		
cumin		28	6 (21.4%)		33.9 ± 14.7		
fennel		25	0	0	0		
caraway		25	0	0	0		
fenugreek		35	13 (37.1%)		63.1 ± 17.2		
dry ginger		36	16 (44.4%)		85.1 ± 19.4		

CIT—Citrinin; nd—not detected; LOD—limit of detection; LOQ—limit of quantification.

**Table 2:** Analytical methodologies for determination of CIT in foods and supplements.

Food/ Supplement	Sample (g)	Extraction Procedure	Clean-Up Procedure	Detection and Quantification	Chromatographic Conditions	LOD (ng/g)	LOQ (ng/g)	References
Rice	20	100 mL of ACN-4% aqueous solution of NaCl (9:1). pH adjusted at 1.5 with undiluted HCl. Shake for 20 min. Filtration through a Whatman No. 4 paper under vacuum.	LLE: n-hexane (100 + 50 mL)/10 min. Addition of 50 mL H_2_O and 50 mL ClCH_3_ to the lower phase. Shaking for 10 min. Collect the lower phase (ClCH_3_). Re-extract twice the upper phase with 25 mL of ClCH_3_. Evaporate ClCH_3_ phase at 40 °C. Add 2 mL MeOH, sonicate, and filter (0.45 μm filter). Evaporate to dryness under nitrogen. For HPLC analysis add 500 μL of MeOH.	HPLC-FD: λ exc. 331 nm λ em. 500 nm	Column: C18 (0.46 × 25 cm, 3 µm) Mobile phase: (0.33 M) H_3_PO_4_/ACN/propan-2-ol (650/400/50). Injection volume: 20 µL Flow rate: 0.5 mL/min RT (min): 15	0.11	0.35	[34]
Rice	2	8 mL of H_2_O. Shake with vortex, for 2 min. 10 mL of ACN containing 5% FAc were added and the tube was vortexed again for 2 min.	QuEChERS: Add 4 g MgSO4, 1 g NaCl, 1 g tri-sodium citrate dehydrate and 0.5 g sodium hydrogen citrate sesquihydrate. Shake by hand (1 min) and by vortex (2 min). Centrifugation at 6500 rpm for 5 min. Dry under N_2_ stream 2 mL of the supernatant. Redissolve with 1 mL MeOH:H_2_O (50:50 *v*/*v*). Filtration through 0.2 μm nylon membrane.	UPLC-FD: λ exc. 331 nm λ em. 500 nm	Column: Zorbax Eclipse Plus RRHD (50 × 2.1 mm, 1.8 µm). Temp: 45 °C Mobile phases: A: H_2_O containing 2% FAc; B: ACN containing 2% FAc. Injection volume: 5 µL Flow rate: 0.5 mL/min	1.5	5.0	[35]
Rice	1	5 mL of MeOH:H_2_O (7:3) in a sonifier, for 5 min. Filtration through a 10 μm filter. Evaporation in a rotary evaporator at 40 °C. Re-dissolution in 5 mL of HEPES buffer (0.1 M, pH 7.5).	Molecularly imprinted solid-phase extraction (m-MISPE) Elution: 1 mL of a methanolic solution of 0.05 M TBA	HPLC-UV-DAD: λ: 331 nm	Column: ACE Excel 2 C18-PFP (100 × 2.1 mm, 2 μm) Temp: 45 °C Flow rate: 0.4 mL/min Mobile phase: ACN:H_2_O (40/60) Injection volume: 100 µL	0.7	2.3	[68]
Red fermented rice (RFR)	0.5–1.0	30 mL of EtOH:H_2_2O (7:3); shake at 200 rpm, for 0.5 h, at 40 °C. Ultrasonication at 40 °C, for 30 min. Shaking on a rotary shaker at 200 rpm for 1.5 h.	Centrifuge all of the above suspension with 3000 rpm/5 min at 25 °C. Filter the supernatant with a 0.45 μm filter.	HPLC-FD: λ exc. 331 nm λ em. 500 nm	Column: Zorbax Eclipse XDB C18 (250 × 4.6 mm, 5 µm) Flow rate: 1.0 mL/min Mobile phase: H_2_O (pH is adjusted to 2.5 with H_3_PO_4_) and ACN (50:50)	-	-	[58]
Red Mold Rice (RMR)	1	10 mL EtOH:H_2_O (75:25) at 80 °C for 30 min with shaking	1 mL suspension is evaporated to dryness in a glass desiccator under vacuum. Add 1 mL ACN. Filtration with a 0.45 µm filter.	HPLC-FD: λ exc. 330 nm λ em. 500 nm LC-MS-ESI	Column: Luna C18 (25 cm × 4.6 mm, 5 µm) Column: Phenomenex Luna C18 Mobile phase: 0.05% TFA in ACN:H_2_O (62.5:37.5) Flow rate: 1.0 mL/min.	-	-	[42]
Red fermented rice (RFR)	1	25 mL MeOH. Vortex for 3 min; Ultrasonication: 30 °C/30 min.	Centrifuge the extraction mixture for 10 min, at 6793× *g*. Pass 1.0 mL supernatant through 0.22 μm filter.	HPLC-FD: λ exc. 330 nm λ em. 500 nm LC-MS/MS: ESI^+^	Column: C18 Waters XTerra RP (4.6 × 250 mm, 5.0 μm); Guard column: Xbridge TM C 18 (4.6 × 20 mm, 5.0 μm); Temp: 30 °C; Mobile phase: acidified H_2_O (pH 2.5 adjusted by H_3_PO_4_) and ACN (*v*/*v*, 65:35); Flow rate: 1 mL/min; RT = 18min; Injection volume: 20 µL Column: Phenomenex Luna C 18 (150 × 2.0 mm, 3.0 μm); Temp: 30 °C; Mobile phase: 90% MeOH–10% H_2_O (containing 2.0 mmol/L NH_4_AC and 0.1% FAc); Flow rate: 0.25 mL/min; RT = 12 min; Injection volume: 5 µL Parent/daughter ions (*m*/*z*): 251/205, 233 (*m*/*z*); Collision energy (CE): 27 and 14 eV	1.0	3.0	[37]
Xuezhikang capsule and *Monascus*-Fermented Products	1.5	10 mL toluene-ethyl acetate- FAc (7:3:1), ultrasonication for 20 min (3 times).	Centrifuge. Evaporate the supernatant. Re-dissolved with 10 mL MeOH and filter through a 0.45 µm membrane filter.	HPLC-FD: λ exc. 331 nm λ em. 500 nm	Column: C18 (250 mm × 4.6 mm i.d., 5 µm); Temp: 25 °C; Mobile phase: A- ACN; B-acidified H_2_O (pH 2.5 adjusted with H_3_PO_4_; Injection volume: 20 µL Flow rate: 1.0 mL/min.	0.187 ng/mL	0.6 ng/mL	[41]
Red yeast rice (RYR) and related products	1	5 mL of MeOH:H_2_O (70:30), for 3 min. Centrifugation at 6000 rpm/20 min; dilute 3 mL of supernatant with a PBS to 30 mL.	Filtration through a microfibre filter. SPE: CitriTest™ IAC Elution: 2 mL of MeOH/0.1% H_3_PO_4_ (70:30).	HPLC-FD: λ exc. 331 nm λ em. 500 nm Confirmation: LC-MS/MS: ESI^+^	Column: Phenomenex Gemini 5u C18 (4.6 mm × 250 mm, 5 µm); Temp: 25 °C; Mobile phase: 0.1% H_3_PO_4_ with H_2_O/ACN (55:45) Flow rate: 1 mL/min; Injection volume: 50 µL; Column: Phenomenex Gemini C 18 (20 mm × 2.00 mm, 3 µm); Temp: 20 °C; Mobile phase: A- 100% H2O; B- 100% ACN both with 0.1% FAc Flow-rate: 0.5 mL/min Injection volume: 10 µL RT (min): 2.18 Parent/daughter ions (*m*/*z*): 251.1/205.1, 233.0 CE: 23 and 36 eV.	0.8	-	[40]
Red yeast rice (RYR)	1	10 mL of MeOH:H_2_O (80:20, *v*/*v*). Vigorous vortex stirring for 3 min. Ultrasound bath at room temperature for 1 h.	Centrifuge at 3000 rpm/10 min, room temperature. Collect and evaporate the supernatant to dryness. Redissolve in 1 mL of MeOH. Filtration through a 0.45 um polyester filter.	MEKC-DAD (Capillary Electrophoresis- micellar electrokinetic capillary chromatography): λ. 216 nm	CE: Uncoated fused-silica capillaries (Agilent) 32.5 cm total length (effective length to detector 24 cm) and 50 µm i. d., with an extended light path of 150 µm.	0.03 µg/mL	0.08 µg/mL	[16]
Grains of red fermented rice Tablets Capsules	1	10 mL of 80% MeOH/60 min, at room temperature, using an ultrasonic bath.	Centrifugation at 3000 g/10 min at 25 °C. The supernatant is filtered through a 0.45 μm Chromafil membrane filter.	LC-DAD-FD-MS^n^ UV: 237 nm FD: λ exc. 331 nm λ em. 500 nm	Column: XBridge C18 (50 × 3.0 mm, 2.5 μm); Temp: 25°C Mobile phase: A—ACN/H2O/FAc (10:90:0.1, *v*/*v*/*v*); B—ACN/H_2_O/FAc (90:10:0.05, *v*/*v*/*v*); Flow rate: 1 mL/min; Injection volume: 5 µL; RT (min): 1.25 ± 0.01	0.0005 μg/mL	0.001 μg/mL	[38]
Wheat grains	25	According to Nguyen et al. [34]	According to Nguyen et al. [34]	HPLC-FD: λ exc. 330 nm λ em. 500 nm	Column: C18 (Spherisorb ODII, 250 × 4 mm, 5 mm); Mobile phase: (0.33 M) H_3_PO_4_/ACN/propan-2-ol (650/400/50); Flow rate: 1 mL/min; Injection volume: 50 µL; RT (min): 5.2	0.25	1.0	[43]
Wheat	100	400 mL ACN/H_2_O/acetic acid (79/20/1, *v*/*v*/*v*). Shaking for 90 min. Centrifugation for 2 min at 3000 rpm.	Transfer 75 μL of supernatant and dried at 40 °C. Reconstitute in 150 μL of ACN/H_2_O (50/50).	Eluent from the HPLC is then split by a 50:50 flow splitter, and 25 μL is injected on the ESI MS/MS.	Column: Gemini C18 (150 × 4.6mm, 5 μm) Guard cartridge: C18, 4 × 3 mm i.d. Mobile phases: A- 5 mM of ammonium formate, and 0.1% FAc in H_2_O. B- 100% ACN Flow rate: 1 mL/min Injection volume: 50 µL; Parent/daughter ions (*m*/*z*): 251.1/ 191.0,205.2,233.1	0.6	1.4	[44]
Corn and wheat	10	50 mL of methenolone (70%) Magnetic stirrer for 30 min. Centrifugation at 4000 rpm/10 min. 1 mL of supernatant was mixed with 49 mL of 10 mM H_3_PO_4_ (pH = 7.5).	Filtration through glass fiber filter paper SPE: CitriTest™ IAC	HPLC-FD: λ exc. 350 nm λ em. 500 nm	Column: C18 (50 × 4.6 mm, 2.5 µm) Temperature: 30 °C Mobile phase: 80% 10mM H_3_PO_4_ (pH = 2.5) and 20% ACN Flow rate: 0.5 mL/min Injection volume: 50 µL RT (min): 2.3		<1	[47]
Maize	5	ACN/H_2_O/acetic acid (79:20:1) in a ratio of 4 mL solvent/g sample. Rotary shaking at 180 rpm, for 90 min. Dilution with an equal volume of ACN/H_2_O/acetic acid, 20:79:1.		LC-MS/MS: ESI According to [69]	Column: C18 (150 × 4.6 mm, 5 μm) Gemini Phenomenex Mobile phase: A- MeOH/H_2_O/acetic acid (10:89:1, *v*/*v*/*v*); B- MeOH/H_2_O/acetic acid (97:2:1) (both phases with 5 mM NH_4_Ac) Flow rate: 1 mL/min Temperature: 25 °C	250		[70]
Cereals and cereal products	20	100 mL DCM and 10 mL 0.5 M H_3_PO_4_. Shaking for 45 min.	Filtration. SPE: Polyamide column (1 g) 50 mL of the extract; Elution: 5 mL 20% FAc in MeOH. Concentration almost to dryness, at 50–55 °C; Redissolve in 1 mL mobile phase. Filtration through a cellulose filter, 0.45 μm	ELISA HPLC-FD: λ ex. 340 nm λ em. 495 nm	Column: LiChrospher 100, RP18, (250 × 3 mm i.d., 5 µm) Temperature: 30 °C; Injection volume 20 µL; Mobile phases: A- 55% MeOH, 10% ethyl acetate, 35% 0.6 M H_3_PO_4_; B- 100% MeOH; Flow rate 0.4 mL/min. RT (min): 6.24–6.33	0.8 (wheat) 2.8 (rye)	3.2 (wheat) 10.3 (rye)	[48]
Cereals: maize, wheat, and rice	5	20 mL MeOH: H_2_O (2:8). Shaking, vigorously, for 15 min. Centrifugation at 8000 *g* for 10 min.	Dilute the supernatant with an equal volume of PBS (NaCl content at 1.6%).	ic-ELISA and lateral-flow ICA strip analyses		Visual: 8 Strip scan reader:1.28–1.86		[71]
Maize	5	20 mL of ACN/H_2_O/acetic acid (79:20:1, *v*/*v*/*v*). Shaking for 90 min with a rotary shaker.	Centrifuge at 3000 rpm, for 2 min. Dilute 350 µL of the extract with the same volume of ACN/H_2_O/acetic acid, 20:79:1.	LC-MS/MS: ESI	Column: Gemini^®^ C 18-column (150 × 4.6 mm i.d., 5 μm) Guard column: 4 × 3 mm i.d. (all from Phenomenex) Temp: 25 °C Mobile phases: A- MeOH/H_2_O/acetic acid (10/89/1, *v*/*v*/*v*); B- MeOH/H_2_O/acetic acid (97/2/1, *v*/*v*/*v*); both containing 5 mM NH_4_AC. Flow rate: 1 mL/min; Injection volume: 5 µL RT (min): 11.8 Precursor ion (*m*/*z*): 251.1; Q3: 233.2/205.21; Collision energy: 25/39	CIT—0.16 DH-CIT—2.0		[46] According to [72,73]
Breakfast cereals	20	20 mL of a 4% KCl at pH 1.5 with H_2_SO_4_. Homogenize and extract with 180 mL ACN for 20 min. Filtration under vacuum.	LLE: n-hexane (100 + 100 mL)/1 min. Add to the lower phase 50 mL H_2_O and 100 mL ClCH_3_. Shake for 10 min. Collect the lower phase (ClCH_3_). Re-extracted three times the upper phase with 20 mL ClCH_3_. Pool the extracts, add 50 mL NaHCO_3_ and shake for 10 min. Collect the upper phase (bicarbonate), acidify to pH 1.5 with HCl and allow to stand about 20 min. Extract 3 times the acidified solution with ClCH_3_ (100, 50 and 50 mL). Pool the ClCH_3_ phases. Evaporate at 40 °C. Redissolve in 2 mL MeOH, sonicate and filter through Spartan 0.2 µm cartridges. Evaporate to dryness under N_2_. Redissolve in 500 µL MeOH.	HPLC-FD: λ exc. 331 nm λ em. 500 nm	Column: C18 spherisorb column (0.46 × 25 cm, 3 µm); Mobile phase: H_3_PO_4_ (0.33 M)/ACN/propan2-ol (700/300/50); Flow rate: 0.7 mL/min RT(min): 19 min.	0.5	1.5	[49]
Complementary foods for infants and young children Breadcrumbs and moldy food samples	5	Homogenize with 20 mL of ACN/H_2_O/acetic acid (79:20:1). Extract for 90 min on a rotary shaker and dilute with the same volume of ACN/H_2_O/acetic acid (79:20:1).	After sedimentation of the diluted extracts by gravity, 5 μL of the diluted extracts are directly injected.	LC-MS/MS: ESI	Column: Gemini^®^ C 18 (150 × 4.6 mm i.d., 5 μm) Temp: 25 °C Mobile phases: A- MeOH/H_2_O/acetic acid (10/89/1); B- MeOH/H_2_O/acetic acid (97/2/1); both with 5 mM NH_4_AC. Flow rate: 1 mL/min; RT (min): 14.56 Precursor/daughter ions (*m*/*z*): 251.0/205.2, 233.0; Collision energy: 25 and 39 eV	30		[1,69]
Apples	50 (whole blended apple)	200 mL ACN: 4% KCl (9:1).	An aliquot of 70 mL is cleaned-up with H_2_O, acidified, evaporated to dryness and redissolved in 1ml CHCl_3_.	TLC λ 366 nm		MDM: 15–20 mg/kg	-	[24,74]
Fruits (apples, oranges, sweet cherries and tomatoes)	5	Dilute with H_2_O to 5 mL. Add 20 mL ACN with 100 mM citric acid. Shake at 150 rpm for 30 min.	Add 2.0 g of NaCl and centrifuge at 10,000 rpm, for 5 min at 10 °C. SPE: MCX+NH_2_ homemade cartridge: pass 4.0 mL of upper ACN layer and collect; evaporate to dryness at 50 °C under N_2_. Reconstitute with 1 mL of ACN/H_2_O (3:7) with 5 mM NH_4_AC. Filter through a 0.22 µm PTFE membrane filter.	UPLC-MS/MS: ESI^+^	Column: C18 ACQUITY CORTECS UPLC (2.1 × 100 mm, 1.6 µm) Temp: 40 °C Mobile phases: A-5 mM NH_4_AC in H_2_O; B- ACN; Flow rate: 0.3 mL/min; Injection volume: 3 µL RT (min): 2.82; Precursor/daughter ions (*m*/*z*): 251.2/205.2,191.1	-	1 ng/mL	[75]
Black olives	25	Blend with 180 mL ACN, 20 mL 4% KCl and 2 mL 20% H_2_SO_4_ for 2 min, at high speed. Filtration.	Add 50 mL hexane. Shake for 15 min. Separate the first 100 mL of the lower phase and add 50 mL CHCl_3_ and 25 mL H_2_O. Collect the lower phase and evaporate to dryness under N_2_ at 55 °C. Redissolve in 1 mL CHCl_3_ and remove the CHCl_3_ under N_2_. Before extracts are spotted, TLC plates are dipped into 10% glycolic acid solution in EtOH for 2 min and then dried for 10 min at 110 °C. The dried toxin extracts are dissolved in CHCl_3_ (100 L and are spotted onto a TLC plate using a micropipette.	TLC-UV: λ 366 nm The plate is developed with toluene: ethyl acetate: ClCH_3_: 90% FAc (70:50:50:20), dried and treated with ammonia vapour for 10–15 s (Martins et al., 2002).		-	-	[52]
Black olives	10 (crushed olive paste)	Add 8 mL of a 4% KCl acidified to pH 1.5 with H_2_SO_4_. Homogenize and extract with 72 mL ACN, for 20 min. Filtration under vacuum.	Add 40 mL n-hexane to the filtrate, shake for 1 min. Discard the upper phase (n-hexane). Repeat this defatting operation twice. Add 20 mL H_2_O and 40 mL CHCl_3_ to the lower phases. Shake for 10 min. Collect the CHCl_3_ phase. Re-extract the upper phase 3 times with 20 mL of CHCl_3_. Pool the 4 CHCl_3_ extracts, extract with 20 mL NaHCO_3_ and shake for 10 min. After separation, acidify the aqueous phase (NaHCO_3_) to pH 1.5 with HCl. Extract the acidified aqueous phase 3 times with CHCl_3_ (40, 10, 10 mL). Evaporate the pooled CHCl_3_ extracts at 40 °C. Add 1 mL, sonicate, and filter through Spartan 0.2 μm cartridges. Evaporate under N_2_. Re-suspended in 500 μL of MeOH.	HPLC-FD: λ exc. 331 nm λ em. 500 nm	Column: C18 nucleosil (0.46 × 25 cm, 4 µm,); Mobile phase: H_3_PO_4_ (0.33 M)/ACN/propanol 2-ol (600/400/50); Flow rate: 0.7 mL/min. RT (min): was about 16 min. Confirmation: another mobile phase in which the amount of H_3_PO_4_ is increased and ACN is decreased as follows H_3_PO_4_ (0.33 M)/ACN/propanol 2-ol (700/300/50) RT (min): 25.0	0.2	0.5	[51]
Lager beer		--	Filter untreated and undiluted beer samples through a 0.45 μm filter. Direct injection of 100 μL into an on-line SPE (fused-core Ascentis Express RP C18)—HPLC system	HPLC-FD λ exc. 335 nm λ em. 497 nm	Column: Phenyl-Hexyl (100×4.6 mm, 2.7 μm) Temp: 50 °C Mobile phases: mixtures of MeOH or ACN with a 0.5% acetic acid in H_2_O, pH 2.8 in the range from 35 to 65%; RT (min): 4.63	0.006 µg/L	0.02 µg/L	[26]
Cheese		ACN-KCl solution (5%; 80:20, *v*/*v*) acidified with H_2_SO_4_ to pH 3. Agitation for 30 min.	Filtration	TLC- fluorodensitome-ter: at 330 nm	Development system: toluene—ethyl acetate–FAc (6/3/1, vol/vol/vol)			[27]
Spices	20 g	ELISA: 100 mL of 70% MeOH; blending for 3 min. Filtration. LC-MS/MS confirmation: 10 g of grinded sample. Mix with 40 mL of ACN:H_2_O (40:10) and vortex vigorously, for 5 min and shake gently for 45 min.	ELISA: 4 mL of extract (supernatant) is transferred through clean-up columns (RIDASCREEN FAST citrinin Assay (6302) for CIT). LC-MS/MS confirmation: Filter the solution through 0.2 μm nylon filter. Dry 2 mL of filtrate under N_2_. Reconstitute in 1 mL of ACN:H_2_O (10:40).	ELISA: 450 nm filter with a differential filter of 630 nm Confirmation: LC-MS/MS: ESI^+^	Column: Hypersil Golden C18 (100 mm × 2.1 mm, 3 μm) Temp: 30 °C Mobile phase: 0.1% FAc in 5mM NH_4_Ac and MeOH Flow rate: 0.3 mL/min Injection volume: 0.5 µL	15		[54]
Fermented meat products	10 g	50 mL of 70% MeOH. Mix at high speed for a minute. Filtration. Dilute 1 mL of filtrate with 49 mL of 10 mM H_3_PO_4_ and mixe. Filter through a microfiber filter.	SPE: IAC column: Load 10 mL of the extract. Wash with 5 mL of 10 mM H_3_PO_4_. Elute with 1 mL of MeOH:10 mM H_3_PO_4_, 70:30. Vortex the eluate and inject.	HPLC-FD: λ exc. 350 nm λ em. 500 nm	Column: Waters Sunfire C18 (4.6 × 20 mm, 2.5 µm); guard column. Mobile phase: H_2_O: 0.1% H_3_PO_4_: ACN (60:40); Flow rate 1.0 mL/min; Injection volume: 50 µL; RT (min): 5 min.	0.5	1.0	[53]
Dry cured ham		ACN–4% KCl aq (9/1, *v*/*v*) acidified by H_2_SO_4_ to pH 3.		TLC- fluorimetric detection at 330 nm	According [27]		20	[64]
Sufu Cooked foods Ham Snacks		Ultrasonic assisted extraction	Centrifugal separation and nitrogen blowing concentration	HPLC-FD λ exc. 331 nm λ em. 500 nm	Column: Agilent Eclipse Plus C 18 Temperature: 28 °C Mobile phase: ACN:H_2_O (35:65) Flow rate: 1 mL/min Injection volume: 10 µL	-	-	[29]

ACN: acetonitrile; ClCH_3_: chloroform; DAD: diode array detector; DCM: dichloromethane; EIA: Enzyme Immune Assays; ELISA: Enzyme-Linked Immunosorbent Assay; ES: extraction solvent; ESI: electrospray ion; EW: ethanol:water); FAc: formic acid; FLD: fluorimetric detector; HCl: hydrochloric acid; Hex: hexane; HEPES: 2-[4-(2-Hydroxyethyl)-1-piperazinyl] ethane sulphonic acid; H_2_O: water; H_2_SO_4_: sulfuric acid; H_3_PO_4_: phosphoric acid; ICA: immunochromatographic assay; ic-ELISA: indirect competitive ELISA; KCl: potassium chlorine; LC-MS/MS: Liquid Chromatography-Mass Spectrometry-Mass Spectrometry; LLE: liquid-liquid extraction; MDM: minimum detectable concentrations; MeOH: methanol; m-MISPE: molecularly imprinted solid-phase extraction; MgSO_4_: magnesium sulphate; NaCl: sodium chloride; NaHCO_3_: sodium bicarbonate; N_2_: nitrogen; NH_4_AC: ammonium acetate; PBS: phosphate-buffered saline; QuEChERs: Quick, Easy, Cheap, Effective, Rugged and Safe; RP: reverse phase; TBA: tetra-n-butylammonium hydrogen sulphate; TFA- trifluoroacetic acid, US: ultrasonication; UV: ultraviolet. --: this step was not been done.
